# A Salutogenic Approach to Understanding the Potential of Green Programs for the Rehabilitation of Young Employees With Burnout: Protocol for a Mixed Method Study on Effectiveness and Effective Elements

**DOI:** 10.2196/15303

**Published:** 2019-10-30

**Authors:** Roald Pijpker, Lenneke Vaandrager, Esther J Veen, Maria A Koelen

**Affiliations:** 1 Health and Society Department of Social Sciences Wageningen University Wageningen Netherlands; 2 Rural Sociology Department of Social Sciences Wageningen University Wageningen Netherlands

**Keywords:** burnout, health promotion, occupational health, program evaluation, rehabilitation, sense of coherence, workforce

## Abstract

**Background:**

Burnout is the leading cause of absenteeism in the Netherlands, with associated sick leave costs amounting to around €1.8 billion. Studies have indicated that burnout complaints increased from almost 14.4% in 2014 to 17.3% in 2018, especially among employees between the ages of 18 and 35 years, and further increases are expected. Although there are many published articles on burnout, not much is known about what constitutes effective rehabilitation (ie, the reduction of burnout complaints and the facilitation of returning to work). At the same time, multiple pilot studies have indicated that green programs are effective in both reducing burnout complaints and facilitating return to work. Green programs have been developed by professionals experienced in using the natural environment to facilitate rehabilitation (eg, through green exercise and healing gardens). The literature nevertheless lacks comprehensive and contextual insight into what works and why.

**Objective:**

The overarching aim of this study is to explore the potential of green programs for young employees with burnout. We present the study protocol from an ongoing research project consisting of 2 phases, each composed of 2 research objectives that sequentially build upon each other.

**Methods:**

The study is based on a sequential design with 4 research objectives, using both qualitative and quantitative research methods. In the first phase, a systematic literature review (research objective 1) and in-depth interviews (research objective 2) will be used to explore mechanisms underlying the rehabilitation of young employees with burnout. In the second phase, a multicase study will be conducted to examine the extent to which green programs are built on mechanisms identified in the first phase (research objective 3). By employing a pretest and posttest design, a specific green program that captures most of those mechanisms will then be evaluated on its effect and process with regard to the rehabilitation of young employees with burnout (research objective 4). The project started in June 2018 and will continue through June 2022.

**Results:**

The first phase (research objectives 1 and 2) is intended to generate information on the mechanisms underlying the rehabilitation of young employees with burnout. The second phase (research objectives 3 and 4) is designed to demonstrate the extent to which and how the selected green program facilitates the rehabilitation of young employees with burnout.

**Conclusions:**

Understanding how green programs can facilitate the rehabilitation of young employees with burnout complaints can help to address this societal issue.

**International Registered Report Identifier (IRRID):**

DERR1-10.2196/15303

## Introduction

### Background

Work-related stress is the leading cause of absenteeism in the Organization for Economic Cooperation and Development countries [1,2], and it is accompanied by significant financial consequences for society [3]. The most significant occupational syndrome, burnout, has been shown to have an adverse effect on the health and well-being of employees (eg, more physical illness) and on the organizations in which they work (eg, less organizational involvement) [4]. In the Netherlands, burnout is the leading cause of absenteeism, with associated sick leave costs amounting to around €1.8 billion [5]. The prevalence of employees with burnout complaints has increased from 14.4% in 2014 to 17.3% in 2018 [6], especially among employees between the ages of 18 and 35 years [7]. Given that organizational development and performance are dependent on the health and well-being of employees [8], it is of utmost importance to address the increase of burnout among young employees.

Burnout is defined as “a work-related state of exhaustion that occurs among employees, which is characterized by extreme tiredness, reduced ability to regulate cognitive and emotional processes, and mental distancing. These four core dimensions of burnout are accompanied by depressed mood as well as by non-specific psychological and psychosomatic distress symptoms” [9]. In general, the development of burnout is fostered through a complex interplay of factors within employees (eg, being a workaholic), factors within the organizational context (eg, excessive workload) [10], and factors beyond the workplace (eg, economic crisis) [11]. Little has been written, however, about the recent increase in burnout complaints among young employees.

The early 1970s witnessed the emergence of a booming burnout industry, consisting of programs focusing on either employees (ie, person-directed interventions) or organizations (ie, organization-directed interventions) [4,12,13]. A further distinction can be made between programs aimed at reducing burnout complaints among employees who are still at work and interventions aimed at facilitating return to work (RTW) among employees who are currently not working because of burnout [4]. Systematic reviews and meta-analyses have focused on either person-directed or organization-directed interventions, indicating that the use of either of these types of interventions alone produces suboptimal results in terms of reducing burnout complaints and facilitating full RTW [14,15]. Burnout programs that combine both person-directed and organization-directed interventions are more likely to be effective in facilitating rehabilitation. Reviews of such programs are nevertheless lacking.

At the same time, a growing body of literature suggests that nature offers opportunities for rehabilitation [16], here defined as developing the ability of young employees to participate and be productive in a sustainable and meaningful way [17]. Studies have demonstrated that experiencing nature can directly enhance physical and mental health and that interacting with natural elements can develop well-being, while offering opportunities for social interaction [18]. These empirical insights are increasingly being translated into “green programs” aimed at facilitating the rehabilitation of employees with burnout [19]. Little is known, however, about the extent to which green programs can facilitate rehabilitation for young employees with burnout.

Typical examples of green programs include green exercise [20] and healing gardens [21]. These programs differ in the *extent* to which nature is used, as well as in the *ways* in which nature is used [22]. One defining characteristic of green programs is that they are provided by professionals who are experienced in using nature to facilitate rehabilitation. Although the first pilot studies indicate that green programs can reduce burnout complaints and facilitate RTW [19,20], not much is known about their effectiveness or underlying mechanisms. The aim of this study is, therefore, to explore the potential of green programs for the rehabilitation of young employees (18-35 years of age) with burnout in the Netherlands.

### Research Objectives

We present the study protocol from an ongoing research project consisting of 2 phases, each proceeding from 2 research objectives that build sequentially upon each other. Before we can understand how green programs could facilitate rehabilitation among young employees with burnout, we must understand the mechanisms underlying such rehabilitation (phase 1). The next logical step is to examine the extent to which green programs are built on those mechanisms and to evaluate a green program on its effect and process for young employees with burnout (phase 2). In line with the overall research objective, the emphasis of this study lies in phase 2. Given the limited knowledge about the rehabilitation of young employees with burnout, however, phase 1 is of utmost importance to the research project.

### Phase 1

#### Research Objective 1

Systematic reviews and meta-analyses have focused on either person-directed or organization-directed interventions, each of which has proved suboptimal in promoting rehabilitation when used exclusively. The first objective of this study is, therefore, to assess the effectiveness of existing combined rehabilitation programs, in addition to examining the mechanisms that influence their effectiveness.

#### Research Objective 2

The increase in burnout complaints among young employees is a recent phenomenon. It is, therefore, necessary to understand how young employees develop and recover from burnout. To this end, the second objective is to understand factors relating to the development of burnout and rehabilitation from burnout among young employees.

### Phase 2

#### Research Objective 3

Although green programs are increasingly assumed to be effective in the rehabilitation of employees with burnout, the extent to which their initiators have built upon the mechanisms identified in phase 1 is unclear. The third objective is, therefore, to describe green programs and examine the extent to which they are built on mechanisms underlying the successful rehabilitation of young employees with burnout.

#### Research Objective 4

To date, there is little understanding concerning the relative effectiveness of green programs in the rehabilitation of young employees with burnout and with regard to the reasons underlying their effectiveness. To address this knowledge gap, the fourth objective of this study is to evaluate the effect and process of a green program for young employees with burnout. The rationale for evaluating only 1 green program is that doing so can ensure the feasibility of the study, with a specific focus on a program that captures most of the mechanisms identified in the first phase.

### Theoretical Framework

This study integrates theories that explain associations between nature and rehabilitation, in addition to drawing on pivotal theories used in health promotion.

### Nature and Rehabilitation

Associations between nature and rehabilitation are assumed to be explained by 4 types of pathways: physiological, mental recovery, social, and psychological [16,18]. The physiological pathway refers to the beneficial effects that being outside and physically active can have on physical well-being or other aspects [18]. The mental recovery pathway alludes to the effects that the “soft fascination” of being in nature can have on acute and chronic stress (eg, improved mood) [18]. The social pathway elicits the positive effects of social contacts that nature may facilitate (eg, a sense of belonging) [18]. The psychological pathway touches on the capacity of nature to serve as a mirror with which to reflect on concrete experiences (eg, through the use of metaphors). These 4 pathways are intertwined. For example, acute or chronic stress can be alleviated by physical activity in the same way that having social contacts can influence reflection [16].

### The Salutogenic Approach

This study adopts the salutogenic approach to investigate how the 4 pathways underlying green programs can facilitate rehabilitation. The salutogenic approach focuses on the processes through which young employees can strengthen (or restore) their capacity to identify and reuse resources within themselves (eg, skills), as well as within their immediate environments (eg, social relations) to deal with stressors (eg, daily hassles) [23]. In this regard, it complements pathogenic strategies aimed at eliminating or alleviating stressors in the workplace. Studies have demonstrated that enhancing resources and people’s capacity to use and reuse those resources can have more sustainable effects as compared with efforts aimed at eliminating stressors [23]. The salutogenic processes underlying this capacity depend primarily on environmental support for certain experiences [23]. The 4 pathways are likely to provide this support. For example, feeling physically and mentally well (ie, the physiological and mental recovery pathways) are inherent resources that enable young employees to deal with stressors. For this reason, this study adopts the salutogenic approach as an overarching framework with which to examine how green programs can support the salutogenic process, thereby facilitating rehabilitation for young employees with burnout.

The resources that can be used to deal with stressors effectively are known as *generalized resistance resources* (GRRs) [24]. The ability to use (or reuse) GRRs is known as the *sense of coherence* (SOC), a global life orientation that represents the extent to which people experience the world as comprehensible, manageable, and meaningful [25]. Studies have indicated that GRRs maintain a reciprocal relationship with SOC. In other words, GRRs predict a high SOC, which in turn enhances the ability to identify and use GRRs [26]. Important GRRs in the workplace include job control, social relations, and task significance [27]. Job control is defined as an employee’s decision-making authority, opportunities to use skills and knowledge, and opportunities to participate [17]. Social relations are defined as the extent to which individuals are able to count on information, assistance, support, and appreciation from their colleagues at work [17]. Task significance is defined as the perception that an individual’s job has a positive impact on other people [17]. Although resources are not GRRs unless an employee can use them [23], this study uses these 3 GRRs and SOC as indicators of rehabilitation among young employees.

Research has consistently demonstrated that employees with a strong SOC experience fewer burnout complaints than do employees with a weak SOC [28]. Understanding the salutogenic process through which the 4 pathways can strengthen the SOC of young employees could, therefore, enhance insight into how green programs can facilitate their rehabilitation. However, little is known about this salutogenic process. It can nevertheless be explained by attribution theory [29], which is intended to explain how and in what way people process information in the attempt to understand events, judge those events, and act on those events [30]. Attribution theory is strongly related to the 3 dimensions of SOC—comprehensibility (understanding events), manageability (acting on events), and meaningfulness (judging events) [30,31]—thus making it possible to study the development of SOC.

**Figure 1:**
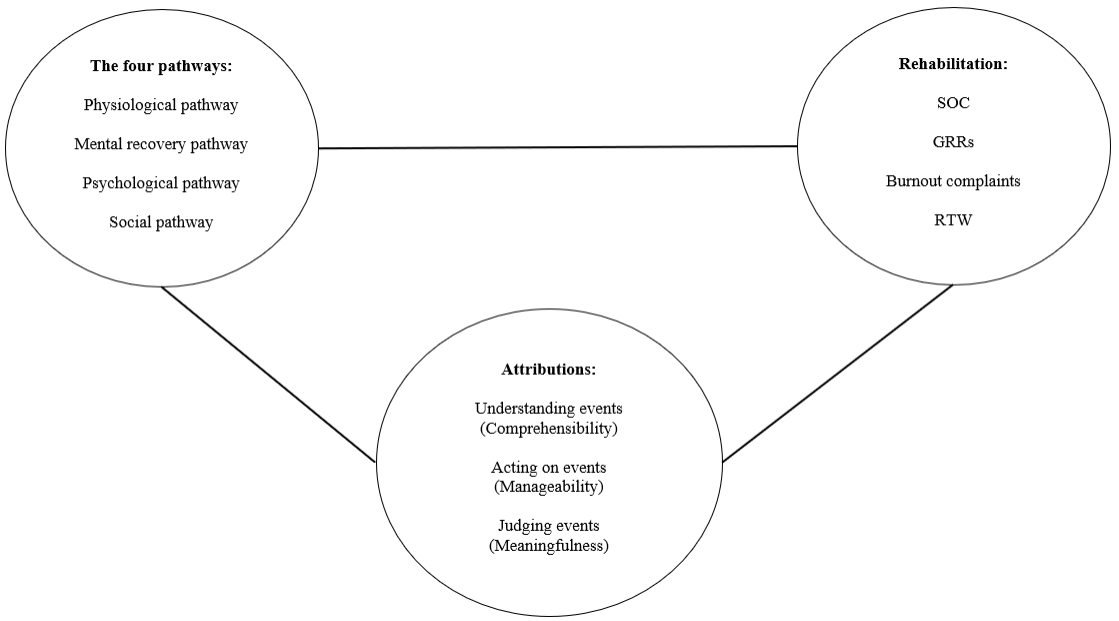
Conceptual framework. SOC: sense of coherence; GRR: generalized resistance resource; RTW: return to work.

Taken together, the aforementioned theories offer a coherent framework with which to explain how green programs can facilitate the rehabilitation of young employees with burnout (see Figure 1). As shown in the figure, the 4 pathways are likely to provide environmental support for certain experiences that can enhance the GRRs and SOC of young employees. For example, feelings of mental and physical well-being are inherently GRRs (mental recovery and physiological pathways), as is having social relations (social pathway). In addition, the psychological pathway can foster an employee’s reflection on positive life experiences, with nature serving as a mirror. By reflecting on such experiences, young employees can shed light on exactly what has happened in terms of stressors and the GRRs at their disposal (comprehensibility), in addition to understanding how they have taken action to use their GRRs (manageability) and making sense of why dealing with those stressors was worthwhile (meaningfulness). This salutogenic process is assumed to enhance both the GRRs and the SOC of young employees, thereby facilitating their rehabilitation.

## Methods

### Study Design

This study is based on a sequential exploratory research design involving mixed method—more specifically, a combination of qualitative and quantitative research methods [32]. In the following sections, the research methods and activities are explained for each research objective.

### Methods and Activities for Phase 1

The first phase is intended to identify mechanisms underlying the rehabilitation of young employees with burnout. First, a systematic review will be conducted to assess the effectiveness of existing combined rehabilitation programs as well as factors underlying their effectiveness (research objective 1). Second, interviews with young employees will be used to investigate factors related to the development of burnout and rehabilitation (research objective 2).

### Research Objective 1

#### Method

A systematic review will be employed according to the guidelines for Reporting Items for Systematic Reviews and Meta-Analyses [33]. Details of the protocol for this systematic review have been registered on PROSPERO [34].

#### Activities

A total of 7 electronic databases are searched: Psychology and Behavioral Sciences Collection, PsycARTICLES, Web of Science (all databases), Scopus, SocINDEX, PubMed, and PsycINFO. Search terms are based on the operationalization of burnout according to the most frequently used questionnaire for measuring burnout—the Maslach Burnout Inventory (MBI)—combined with *intervention*. The inclusion period will be specified from 1970 to 2019. No other electronic search strategies will be employed, as the literature review will not include gray literature and non–peer reviewed publications. The reference lists of the articles reviewed and the systematic reviews identified will also be searched for additional relevant studies.

A total of 5 inclusion and exclusion criteria will be applied to the studies identified through the search. First, only interventions that combined both person-directed and organization-directed approaches (eg, cognitive-based therapy with interventions in the workplace) will be included. Second, studies that do not use the MBI to measure burnout will be excluded. Third, only experimental study designs will be included (eg, cross-sectional studies will be excluded). Fourth, only studies focusing on employees will be included. Finally, only studies reported in English will be included.

We will use a descriptive narrative synthesis of the effects of the programs on burnout or RTW that are included using summary data published in the articles. In addition, a detailed description of each study will be compiled, including participant characteristics, the theory and approach used, levels targeted, program content, and the duration and intensity of the program. All researchers will be involved in synthesizing the data and the resolution of any discrepancies.

### Research Objective 2

#### Method

A total of 20 open semistructured interviews will be conducted with young employees who have either been diagnosed with burnout or have successfully recovered from a diagnosed burnout. If needed, additional interviews will be conducted until data saturation is achieved. For the purposes of this study, successful recovery will be defined as full RTW, whether with the employee’s current employer or with a new employer. A narrative inquiry approach will be used to investigate the mechanisms underlying the development of burnout in young employees and how their rehabilitation proceeded. Narrative inquiry is defined as systematically listening to people’s life stories [35]. These stories will be elicited through timelines—an established research tool involving drawing and visually exploring life experiences [36]. The drawings and visually explored life experiences will then be used to structure the open semistructured interviews. For example, participants will discuss and explain their timelines chronologically and describe life events and turning points with regard to the development of burnout and subsequent rehabilitation.

#### Activities

We will recruit young employees (18-35 years of age) who have either been diagnosed with burnout or have successfully recovered from a diagnosed burnout. As a criterion for inclusion, an employee’s diagnosis must have been made by an occupational physician or general practitioner. As a criterion for exclusion, the burnout must not have been a direct consequence of a psychiatric disorder (eg, clinical depression), which can be ascertained according to the official process for diagnosing burnout in the Netherlands [37]. Participants will be recruited through social media (eg, Twitter and Facebook) and the distribution of flyers about the study in the offices of occupational physicians and general practitioners, thereby enabling employees to contact the researchers directly for additional information concerning participation in the study.

Atlas.ti 8 (Windows) software will be used to analyze the data. All researchers will code the transcriptions of the qualitative data. Interview transcripts will be analyzed using interpretative phenomenological analysis, which takes the world of participants into account and analyzes the articulation of events, processes, and relationships [38]. Any lack of congruence will be discussed until an agreement is reached. Particular attention will be given to (1) stressors, (2) attributions, (3) GRRs, and (4) SOC.

### Methods and Activities for Phase 2

The second phase is intended to examine the extent to which green programs are built on mechanisms identified in the first phase (research objective 3) and to evaluate a green program on its effect and process for young employees with burnout (research objective 4).

### Research Objective 3

#### Method

A multiple case study will be used [39] to pursue this objective. Focus group interviews with the initiators of green programs will be used to collect data contributing to the description of the green programs, complemented by individual open semistructured interviews with initiators.

#### Activities

An inventory of green programs available in the Netherlands will be compiled in collaboration with several stakeholders involved in this project. As criteria for inclusion, a green program must target young employees with burnout and have been developed by practitioners with ample experience using nature to facilitate rehabilitation.

Using a purposive sampling strategy, a heterogeneous sample of a maximum of 10 green programs will be selected. Focus group interviews with the initiators of green programs will be used to describe the programs, focusing on the following characteristics: underlying theoretical assumptions, methodologies used, program aims and content, processes, outcome measures, and follow-up. This information will be complemented by individual open semistructured interviews with each of the same initiators.

Atlas.ti 8 (Windows) software will be used to analyze the data. The transcriptions of qualitative data will be coded by all researchers, informed by the mechanisms underlying rehabilitation, as identified in research objectives 1 and 2. First, a within-case analysis [38] will be conducted to obtain a thorough description of the development of the selected green programs. Second, a cross-case analysis [39] will be conducted to develop a comprehensive description of green programs and the extent to which they are built on the mechanisms underlying the rehabilitation of young employees with burnout, as identified in research objectives 1 and 2.

### Research Objective 4

#### Method

A pretest and posttest design will be used to conduct an effect and process evaluation of an existing green program aimed at the rehabilitation of young employees with burnout [29]. A pretest (T0) measurement will be taken at the start of the green program, with the posttest (T1) measurement being taken after the program has ended. This information will be supplemented with an additional follow-up (T2) measurement. The emphasis of this study is on understanding the extent to which the green program to be selected is effective in promoting the rehabilitation of young employees, in addition to considering how and why the program did or did not work. The next logical step would be to compare the effect and process of this program with those of another rehabilitation program, but doing so would be beyond the scope of this study.

Although the selection of the green program will depend on the mechanisms identified in phase 1 and on the expert advice provided by the advisory board (see section Discussion), the following 3 criteria have been predefined. First, the green program should be officially registered with the Dutch Association for Green Care Professionals [40]. In other words, it should be conducted by a registered coach or therapist with specific expertise in using nature to facilitate rehabilitation. Second, the green program should take place outdoors. In other words, it should involve allowing the participants to experience nature (eg, walking outside while being coached) and to interact with its elements (eg, shaping nature). For example, placing plants in the offices of young employees would not be considered as a green program. Finally, the duration and frequency of the green program should be substantial. For example, a green program that offers a single walk outdoors would not be eligible.

The experimental and control groups will consist of young employees with either burnout complaints or a diagnosed burnout, who will then participate in the green program to be selected. The rationale for including young employees with burnout complaints, rather than only employees who are diagnosed with burnout, is to ensure that the number of participants included will be sufficient to measure the effects on their rehabilitation. Participants in the experimental group will participate in the green program to be selected, whereas the participants in the control will not be enrolled in a green program. However, it is likely that young employees in both groups will take action by themselves to cope with their burnout complaints or diagnosed burnout. Therefore, this study will examine those possible coping strategies in both groups (see the following section Activities) to better understand the effect and process of the green program.

To determine the number of participants in the experimental and control groups, formal sample size calculations will be employed using G*Power, version 3.0.10 based on 1 of the outcomes of rehabilitation: SOC, GRRs, RTW, or burnout complaints. The outcome used for the power calculation and the exact research design will be based on research objectives 1, 2, and 3.

#### Activities

Young employees who have been diagnosed with burnout (as explained in research objective 2) will be recruited through physicians/general practitioners, as these employees are not currently working because of burnout. The recruitment of young employees with burnout complaints who are currently still working will be done in a similar manner (eg, through social media and by placing flyers about the study in the offices of occupational physicians and general practitioners). In addition, employees will be approached through organizational newsletters, to recruit young employees with burnout complaints who do not use social media or contact their occupational physicians or general practitioners about their complaints.

The Burnout Assessment Tool (BAT) (33 items) is a reliable, validated Dutch questionnaire for measuring burnout complaints [9]. Although the MBI is the most frequently used questionnaire, it is subject to several conceptual, technical, and practical imperfections [9]. In contrast, the BAT is assumed to be more versatile, and it can even be used to assess and monitor employees who are currently not working (eg, within the context of RTW programs) [9]. Moreover, psychometric studies have demonstrated that the total score on the BAT can be used as an indicator of burnout [9].

The concept of SOC is easily applicable to workplaces. It will be measured using the Dutch version of the Orientation to Life Questionnaire (13 items) [41]. Given that SOC represents a global life orientation shaped by its 3 dimensions, the total SOC score will be calculated [40].

The Dutch versions of the Knowledge Intensive Working Environment Survey Target and the second version of the Copenhagen Psychosocial Questionnaire II have been validated to measure the 3 GRRs: job control (16 items), social relations (12 items), and task significance (3 items) [42,43]. Each of these questionnaires assesses a broad range of psychosocial work factors, and neither is attached to any specific theory or model. The various subscales will be combined into 3 latent variables [26,27].

The Causal Dimension Scale II (12 items) will be used to measure the attribution styles of young employees, operationalized according to 4 dimensions: locus of causality, stability, personal control, and external control [31]. As the questionnaire has not been validated in Dutch, it will be translated according to the cross-cultural adaption process [44].

RTW will be operationalized as the number of days until RTW or full RTW at follow-up.

The questionnaires selected for the effect evaluation are listed in Table 1. In addition to the instruments mentioned in the table, demographic information will be obtained through items concerning age, sex, country of birth, the highest level of education completed, and current (or previous) job. These data will be collected only at the T0 stage.

With regard to the effect evaluation, quantitative data will be assessed, particularly concerning the potential for bias because of nonresponse, the extent and pattern of missing data, and heterogeneity between groups [45]. The data will, therefore, be checked to determine whether nonresponse was related to gender, age, or other demographic variables, as well as whether any such associations could explain differences in response rates among the groups. The next step will involve assessing the psychometric properties of the data obtained by the instruments listed in Table 1 (eg, internal consistency, based on Cronbach alpha) and comparing them with the properties reported by their developers. The analyses will be performed using the IBM SPSS Statistics 24 software, based on descriptive statistics (eg, means, frequencies, and 1-way repeated-measures multivariate analysis of variance) [45].

**Table 1:** Questionnaires to be used for the effect evaluation.

Questionnaire	Captures	Domains	Likert scale
Burnout Assessment Tool [9]	Burnout complaints	Exhaustion Mental distance Emotional impairment Cognitive impairment Depressed mood	1-5
Sense of coherence—Orientation to Life Questionnaire [41]	Comprehensibility, manageability, meaningfulness	Manageability Comprehensibility Meaningfulness	1-7
Knowledge Intensive Working Environment Survey Target (KIWEST)/Copenhagen Psychosocial Questionnaire II (COPSOQ II) [42,43]	Job control (GRR^a^)	Influence on work Possibilities for development Job autonomy Illegitimate tasks	1-5
KIWEST/COPSOQ II [42,43]	Social relations (GRR)	Social support from supervisors Social support from colleagues Rewards Social community at work	1-5
KIWEST/COPSOQ II [42,43]	Task significance (GRR)	Meaning of work	1-5
Revised Causal Dimension Scale II [31]	Attribution style	Locus of causality Stability Personal control External control	1-9

^a^GRR: generalized resistance resource.

To better understand the extent to which the green program (to be selected) is effective in facilitating the rehabilitation of young employees with burnout, a process evaluation will be performed using open semistructured interviews with the study participants and initiators of green programs. The interviews will emphasize how the program has been executed, as well as mechanisms underlying how and why the program (see Figure 1) worked in relation to rehabilitation. Interviews will also be conducted with the study participants in the control group to explore how they have coped with their burnout complaints or diagnoses.

For the process evaluation, the open semistructured interviews will be recorded and transcribed verbatim. Atlas.ti 8 software will be used to explore the perceptions of the participants with regard to the program and pathways. Any lack of congruence will be discussed with all researchers until an agreement is reached. The rationale for the process evaluation is that it will provide comprehensive and contextual insight into what works and why. This insight will be further enhanced by examining how other possible coping strategies of young employees in both experimental and control groups could have contributed to their rehabilitation.

## Results

The study protocol was reviewed and approved by the Research Assessment Committee from the Wageningen School of Social Sciences, Wageningen University and Research (Multimedia Appendix 1). The research project started in June 2018, and will continue through June 2022. This study protocol includes the methods and activities of 4 different studies that will build sequentially on each other. The first phase (research objectives 1 and 2) is intended to generate information on the mechanisms underlying the rehabilitation of young employees with burnout. The second phase (research objectives 3 and 4) is designed to demonstrate the extent to which and how the selected green program facilitates the rehabilitation of young employees with burnout. The first results are expected to be submitted for publication in 2020.

According to Dutch law, the research project requires formal ethical approval by the Social Sciences Ethics Committee (Wageningen University & Research). This approval was obtained on June 13, 2019. The activities associated with the fourth research objective require consent from the Medical Ethical Committee of Wageningen University, which will be requested during the third year of the project (2020/2021). It is not possible to apply for approval for this research objective at this stage of the project, as it builds on the information obtained through the other 3 research objectives. The required details of the experimental design for the fourth research objective are thus not yet available.

At every stage of the research, participants will be informed about the purpose and content of the study. Moreover, participation will always be voluntary, and participants will be able to withdraw from research activities for any reason at any time. Data confidentiality will be ensured by removing all personal information of the participants from the dataset.

## Discussion

According to the Dutch Public Health Foresight Study, it is important to address burnout among young employees, as it is expected to increase further [46]. By examining mechanisms underlying the rehabilitation of young employees with burnout (phase 1) and examining whether and how green programs facilitate rehabilitation among young employees (phase 2), this study makes a direct contribution to addressing this societal problem.

Given that the BAT has not yet been used in evaluation research on burnout interventions, the systematic review (research objective 1) will be based on the MBI, as it continues to be regarded as the golden standard for measuring burnout. The effectiveness of the green program will be assessed according to the BAT, however, and this will also offer an opportunity to reflect on the validity and reliability of this instrument in intervention research.

A multicase, multi-method design is proposed, including several green programs, using both qualitative and quantitative measures. Rather than examining pathways underlying green programs in isolation, this study adopts a salutogenic approach to exploring the interrelatedness between pathways. This will make it possible to examine the salutogenic mechanisms through which green programs provide environmental support on certain experiences that strengthen young employees’ GRRs and SOC, thereby enhancing insight into how to facilitate their rehabilitation.

Although the salutogenic approach takes the specific work contexts of employees into account, green programs often do not target their daily work environment. The authors acknowledge that improving the working conditions and working environments of young employees is also important to facilitate their rehabilitation. In addition, it would be good to have green programs that could combine their approach with workplace interventions. Given the emphasis of this research project on exploring the potential of green programs for the rehabilitation of young employees with burnout, however, the evaluation of workplace interventions would exceed the scope of this study.

Finally, to enhance the feasibility of this study, an advisory board has been established, consisting of experts from multiple institutions [47]. The overarching aim of the advisory board is to discuss the research project in terms of both content (eg, the research design for research objective 4) and process (eg, the recruitment of participants for research objective 2). Meetings will be organized at least twice a year, thereby allowing the researchers to anticipate and address problems that could threaten the feasibility of the project.

## Appendix

Multimedia Appendix 1 Peer-reviewer report from the Wageningen School of Social Sciences.

## Abbreviations

BAT: Burnout Assessment Tool
GRR: generalized resistance resource
MBI: Maslach Burnout Inventory
RTW: return to work
SOC: sense of coherence
WUR: Wageningen University and Research
