# Supplementary material for: A Salutogenic Approach to Understanding the Potential of Green Programs for the Rehabilitation of Young Employees With Burnout: Protocol for a Mixed Method Study on Effectiveness and Effective Elements
Source: JMIR Res Protoc. 2019 Oct 30;8(10):e15303. doi: 10.2196/15303 (PMC6913693; doi:10.2196/15303)
Supplement: Multimedia Appendix 1 [file resprot_v8i10e15303_app1.pdf]

Review of PhD project "The potential of green programmes for the rehabilitation of young employees with burnout: a salutogenic approach" from the Health and Society Group and the Rural Sociology Group of Wageningen University [RP-MK1]

Please, rate each aspect on a four-point scale between outstanding and poor by ticking the appropriate box. Please also explain your evaluation and give comments and suggestions how to improve the project.

**Quality**	**Outstanding**	**Good**	**Fair/some weaknesses**	**Poor**
Clarity of the proposal	☐	☒	☐	☐
Originality of the proposal	☐	☒	☐	☐
Use of existing knowledge	☐	☒	☐	☐
Methodological approach	☒	☐	☐	☐
Scientific relevance	☒	☐	☐	☐

Please, provide detailed comments in support of the rating you have given above; please, also add comments that may help the applicant to improve the proposal if necessary

Comments:
The problem description identifies the individual level interventions of rehabilitation programmes as a problem, but the research still focuses at the level of the individual in its efforts to understand the causes of burnout and pathways to rehabilitation. Perhaps a broader contextual analysis is also needed? I.e. to put this development within a more critical framework; what societal structures may be leading to an increase in burnout amongst younger people and how could that be addressed, e.g. could it be related to the increasingly precarious nature of employment which affects young people disproportionally? Such socio-economic factors are hinted at in the proposal, but only treated as external 'stressors' that individuals need to be able to cope with. From my perspective, a broader sociological analysis is missing from the project proposal. In this vein, I would have liked to see a more critical perspective on green programmes, i.e. there is an assumption here that 'nature' is universally beneficial as a site for rehabilitation, so this is what I recommend as a minor revision of the proposal. There is a large literature in health geography on 'therapeutic landscapes' which may provide some input into the experiential diversity of environments that are positioned as health promoting or healing, but also a greater emphasis on contextualizing these within contemporary debates on health/wellbeing. This being said, the research is timely and will make an important contribution on an emerging, but pressing societal problem.

**Feasibility**	**Outstanding**	**Good**	**Fair/some weaknesses**	**Poor**
Feasibility of the work plan	☐	☒	☐	☐
Appropriateness of the scope of the research	☒	☐	☐	☐
Expertise in the supervision team	☒	☐	☐	☐
Cooperation with others	☐	☒	☐	☐

Please, provide detailed comments in support of the rating you have given above; please, also add comments that may help the applicant to improve the proposal if necessary

Comments:<br>The scope of the research is comprehensive and well considered and the cooperation with external actors looks appropriate. The research can be successfully guided by the relevant knowledge and research expertise provided by the supervisory team.<br>The time allocated for writing the systematic review paper in year one seems underestimated, especially as it is done at the same time as rather a lot of TSP activities, and education and supervision duties. However, overall the work plan seems feasible.

**Societal relevance and expected societal impact**	**Outstanding**	**Good**	**Fair/some weaknesses**	**Poor**
	☒	☐	☐	☐

Please, provide detailed comments in support of the rating you have given above; please, also add comments that may help the applicant to improve the proposal if necessary

Comments:<br>it is particularly nice that workshops are built in as a pathway to societal impact throughout the course of the project.

**Ethical issues**	**Yes**	**No**
Do you find ethical issues present in this proposal?	☒	☐
If yes, are the ethical issues handled satisfactorily?	☒	☐

Please, provide detailed comments that may help the applicant to improve the proposal if necessary

Comments:

**Overall assessment**		
Do you recommend revising the proposal?	☒ **Yes**	☐ **No**
If yes, which kind of revision do you recommend?	☐ **Major**	☒ **Minor**

Thank you very much for you co-operation and please return within two weeks to Wageningen School of Social Sciences: e-mail to: wass@wur.nl

GUIDELINES for the review of a PhD project proposal

QUALITY AND RELEVANCE	
**Clarity of the proposal**	Are the objectives, scientific and social relevance, hypotheses, methodology and work plan clearly described?
**Originality**	Does the proposal show high originality as should be expected from a PhD project in terms of the questions asked and/or the method applied?
**Use of existing knowledge**	Does the research plan show that excellent use has been and will be made of existing and relevant knowledge and expertise?
**Methodological approach**	Is the methodological approach clearly linked to and appropriate for the research questions, and is it adequate?
**Scientific relevance**	Is the proposed research scientifically relevant in terms of its contribution to (inter)disciplinary knowledge and understanding?
**Societal relevance**	Does the proposed research address societally relevant questions or issues?
FEASIBILITY	
**Scope of research**	Is the scope of the research project appropriate to the ambitions?
**Work plan**	Can the proposed research be finished in the period mentioned? The period includes writing the thesis but not necessarily the defence. Take into account that a PhD will spend approximately 75% of the time on thesis research.
**Expertise in supervision**	Is the supervision qualitatively and quantitatively adequate?
**Co-operation with others**	Is the envisaged co-operation with others adequate, or have any necessary institutions been left out?
ETHICAL ISSUES	
**Presence**	Please indicate if the proposed research will confront ethical issues that may be related to conflicts of interest between funders and researchers, to the interests and safety of participants, to the security of data and to the security of the researchers.
**Handled satisfactorily**	Please indicate if the proposal sufficiently addresses the major issues as described in further detail in the appendix: i.e. conflicts of interest; recruitment of participants; consent by participants; informed consent form; security of data; secondary data collection; research risks; research in unsafe areas.
OVERALL ASSESSMENT	
**Recommendations**	Would you recommend revisions?<br>If so, would you recommend minor or major revision(s) of the project proposal?

Review of PhD project "The potential of green programmes for the rehabilitation of young employees with burnout: a salutogenic approach" from the Health and Society Group and the Rural Sociology Group of Wageningen University [RP-MK2]

Please, rate each aspect on a four-point scale between outstanding and poor by ticking the appropriate box. Please also explain your evaluation and give comments and suggestions how to improve the project.

**Quality**	**Outstanding**	**Good**	**Fair/some weaknesses**	**Poor**
Clarity of the proposal	☐	☒	☐	☐
Originality of the proposal	☒	☐	☐	☐
Use of existing knowledge	☐	☒	☒	☐
Methodological approach	☐	☒	☐	☐
Scientific relevance	☐	☒	☐	☐

Please, provide detailed comments in support of the rating you have given above; please, also add comments that may help the applicant to improve the proposal if necessary

Comments:
First of all, I would like to compliment the PhD candidate on this original proposal, which aims to tackle a relevant health issue in today's society.

I would recommend to make more use of the burnout literature to date, and more specifically of the JD-R model (or JD-R approach) as this provides – albeit oversimplified and limited in other ways of course- concrete insight in the complexity and multidimensionality of the problem.

Furthermore, as currently presented, the green programmes still seem to be an individual approach to burnout. Of course in the green programme, the burnout employee experiences – temporarily- a different context both social and physical, but return to work seems to be the endpoint and the work context itself does not change. In other words, it seems that only the individual is expected to change (and learn skills?). It seems that this is not entirely in line with the aforementioned complexity and multidimensionality of burnout, in which also work context (e.g. job demands or work stressors) play an important role. This should be clarified some more in the proposal.

For objective 1, I would recommend to assess participation (rate) of the existing programmes. Is this reported? If yes, who participates? (And who does not?) What is known about participation of young employees (or age groups in general)? Participation is key to effectiveness of course, and insight in this could inform the evaluation of the process under objective 4.

Objective 4 needs some clarification.
- Are the 5 different green programmes already existing?
- It is stated that the process of green programmes will be evaluated. What is meant with that? The process of implementation? Will a framework (or something like that) be used for this evaluation?
- Is effectiveness compared to treatment as usual? In that case: what is treatment as usual? Are employees that receive treatment as usual also included in the study? What is recovery time? What are current NVAB guidelines for burnout?

One last remark is that the problem and the green programmes seem to be for employees in general. It is not really clear what makes the case for young employees different from that of older employees (other than age). In other words: what could explain the higher prevalence of burnout among young employees?

**Feasibility**	**Outstanding**	**Good**	**Fair/some weaknesses**	**Poor**
Feasibility of the work plan	☐	☒	☐	☐
Appropriateness of the scope of the research	☐	☒	☐	☐
Expertise in the supervision team	☐	☒	☒	☐
Cooperation with others	☐	☒	☐	☐

Please, provide detailed comments in support of the rating you have given above; please, also add comments that may help the applicant to improve the proposal if necessary

Comments:
Feasibility of the project in general seems good to me.

I would suggest that an expert on burnout (or maybe more general on work and organizational psychology) would be of added value. Maybe an expert could be consulted for parts of the study (objective 2, 3 and 4)?

**Societal relevance and expected societal impact**	**Outstanding**	**Good**	**Fair/some weaknesses**	**Poor**
	☒	☒	☐	☐

Please, provide detailed comments in support of the rating you have given above; please, also add comments that may help the applicant to improve the proposal if necessary

Comments:

This project targets a contemporary health issue, which is expected to grow even further in the near future. Therefore, I feel this project is certainly warranted.

I strongly support the development of guidelines or tools for occupational health professionals. I would suggest to seek input among these professionals when developing these.

Stating that green programmes will likely be cost-effective (reduce sick-leave costs etc) when you are not actually performing a CEA is a bit of a stretch. Furthermore, stating that ‘absenteeism rates are likely to reduce in the long term’ is a very bold statement. It is not clear what type of absenteeism is meant here.

**Ethical issues**	**Yes**	**No**
Do you find ethical issues present in this proposal?	☒	☐
If yes, are the ethical issues handled satisfactorily?	☒	☐

Please, provide detailed comments that may help the applicant to improve the proposal if necessary

Comments:<br>It appears that the PhD candidate sufficiently assessed ethical issues. Planned actions for ethical review seem adequate.

**Overall assessment**		
Do you recommend revising the proposal?	☒ **Yes**	☐ **No**
If yes, which kind of revision do you recommend?	☐ **Major**	☒ **Minor**

Thank you very much for you co-operation and please return within two weeks to Wageningen School of Social Sciences: e-mail to: wass@wur.nl

GUIDELINES for the review of a PhD project proposal

QUALITY AND RELEVANCE	
**Clarity of the proposal**	Are the objectives, scientific and social relevance, hypotheses, methodology and work plan clearly described?
**Originality**	Does the proposal show high originality as should be expected from a PhD project in terms of the questions asked and/or the method applied?
**Use of existing knowledge**	Does the research plan show that excellent use has been and will be made of existing and relevant knowledge and expertise?
**Methodological approach**	Is the methodological approach clearly linked to and appropriate for the research questions, and is it adequate?
**Scientific relevance**	Is the proposed research scientifically relevant in terms of its contribution to (inter)disciplinary knowledge and understanding?
**Societal relevance**	Does the proposed research address societally relevant questions or issues?
FEASIBILITY	
**Scope of research**	Is the scope of the research project appropriate to the ambitions?
**Work plan**	Can the proposed research be finished in the period mentioned? The period includes writing the thesis but not necessarily the defence. Take into account that a PhD will spend approximately 75% of the time on thesis research.
**Expertise in supervision**	Is the supervision qualitatively and quantitatively adequate?
**Co-operation with others**	Is the envisaged co-operation with others adequate, or have any necessary institutions been left out?
ETHICAL ISSUES	
**Presence**	Please indicate if the proposed research will confront ethical issues that may be related to conflicts of interest between funders and researchers, to the interests and safety of participants, to the security of data and to the security of the researchers.
**Handled satisfactorily**	Please indicate if the proposal sufficiently addresses the major issues as described in further detail in the appendix: i.e. conflicts of interest; recruitment of participants; consent by participants; informed consent form; security of data; secondary data collection; research risks; research in unsafe areas.
OVERALL ASSESSMENT	
**Recommendations**	Would you recommend revisions?<br>If so, would you recommend minor or major revision(s) of the project proposal?
